# Three‐Dimensional Printing‐Assisted Masquelet Technique in the Treatment of Calcaneal Defects

**DOI:** 10.1111/os.12873

**Published:** 2021-03-25

**Authors:** Laifu Zhang, Chengyin Lu, Yaqing Lv, Xiaohui Wang, Shaoyong Guo, Hailong Zhang

**Affiliations:** ^1^ Henan University of Traditional Chinese Medicine Zhengzhou Henan China; ^2^ Luoyang Orthopedic‐Traumatological Hospital of Henan Province (Henan Provincial Orthopedic Hospital) Luoyang Henan P.R. China

**Keywords:** Bones, Bone diseases, Bone transplantation, Calcaneus, Fracture healing, Models, Printing, Reconstructive Surgical procedures, Three‐dimensional

## Abstract

**Objective:**

The aim of the present study was to summarize the clinical efficacy of three‐dimensional (3D) printing technology combined with the Masquelet technique in the treatment of calcaneal defects.

**Methods:**

From January 2018 to April 2019, 3D printing combined with induced masquelet technology was used to treat four patients with calcaneal defects, including two men and two women. The patients were aged 22–52 years old, with an average age of 36 years. There were two cases of traffic accident injuries, there was one case of a fall from height, and there was one case of crush injury. CT scans were used to reconstruct the bilateral calcaneus, mirror technology was used to construct the bone defect area, and Materialise 3‐matic software was used to design the calcaneus shaper mold and 3D print the mold. During the operation, the mold was used to shape the bone cement and fill the bone defect. In the second stage, the bone cement was removed and autologous bone was implanted to repair the bone defect. All patients were followed up to observe the effect.

**Results:**

All four patients were followed up for 14 months (range, 10–18 months). There were three cases of infectious bone defects: two cases of *Escherichia coli* and one case of *Pseudomonas aeruginosa*. The 3D printed mold was used to shape the bone cement. During the operation, it was found to have a high degree of matching with the defect area of calcaneus. There is no need to adjust it again, and the wound healed well after the first stage. In the second stage of surgery, it was found that the induced membrane formed was complete and of appropriate size; the bone cement was easily removed during the operation. The fracture healing time was 3–6 months, with an average of 4 months. At the last follow up, there was no pain and the patients walked with full weight bearing. The Maryland score was 94 points (range, 88–98 points); three cases were excellent and one case was good. The AOFAS score ranged from 86 to 98, with an average of 92.8 points; three cases were excellent and one case was good.

**Conclusion:**

Three‐dimensional printing technology combined with induced membrane technology is an effective approach for treating calcaneal bone defects.

## Graphical abstract

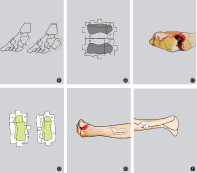

a. 3D printed model of the affected side and healthy side. b. Mold design of the shaper. c. Debride the wound to the osteotomy surface and see the seepage of fresh blood. d. The shaper mold will solidify the bone cement. e. The lateral skin flap of the leg repairs the skin defect of the foot. f. Appearance of the last follow‐up.

## Introduction

The calcaneus is an important structure that is vital for the stability and function of the foot. Calcaneus defects can result from trauma, infection, and skin defects. In the treatment of such defects, problems arise such as large surgical trauma, long cycle, poor healing of fracture sites, and many complications. At present, the most common treatment methods for calcaneus defects are autogenous bone (or musculocutaneous flap) transplantation technology with blood vessels, Ilizarov bone transfer technology, and Masquelet induced membrane technology. Vascular bone (or musculocutaneous flap) transplantation technology involves transplanting a vascularly pedicled autologous bone flap (or musculocutaneous flap) into the bone defect area and anastomosing the blood vessels. The bone defect is then repaired through bone healing and shaping processes. Using this approach, bone defects can be repaired at one time and the bone heals quickly^1^. However, the anastomosis blood vessels need to be accurately separated during the operation, which requires the surgeon to be proficient in microsurgery technology. When the surgery fails, amputation is usually necessary. The microsurgery technology has a steep learning curve, which limits the application of this technology^2^.

Ilizarov bone transfer technology^3^ uses the principle of distraction osteogenesis to repair bone and soft tissue defects by adjusting the position and angle of the external fixator under the action of tension–stress stimulation through the bone's ability to repair itself. This technique has a wide range of indications and is especially suitable for patients with soft tissue defects and infections. However, due to the irregular shape of the calcaneus and the treatment cycle of this technique, there is a positive correlation with the size of the bone defect. When there are many bone defects, the healing time will be prolonged, and the difficulty of postoperative care will be increased. Prolonged fixation will also bring about nail channel infection, fixation loosening, joint adhesion stiffness, nerve damage, and many other problems^4^.

Masquelet technology, also known as induced membrane technology, was carried out in two stages in the repair of bone defects. In the first stage, after thorough debridement of the fracture site, a suitable method was selected for fixation, and a spacer (commonly used polymethyl methacrylate) was placed in the bone defect area to induce the formation of a fibrous membrane. For the second stage, after several weeks, once the induced membrane had formed, the induced membrane was cut, and the implant was removed. The induced membrane was retained, and autologous bone or a mixture of autologous bone and bone substitute material was implanted in the induced membrane to close the induced membrane. Fixed for several weeks to promote the formation of new bone, thereby repairing bone defects. Studies have shown that the induced membrane has a rich vascular system and contains a variety of growth factors, such as VEGF and BMP‐2, which can promote the proliferation and differentiation of osteoblasts, and accelerate the process of bone mineral density and bone stiffness. It can also prevent the resorption of bone graft and promote bone ingrowth to repair bone defects, which is highly similar to autologous periosteum^5^, ^6^, ^7^. Due to the wide Masquelet adaptation technique, the relatively simple operation, and the length of treatment time not being limited by the size of the defect, it has become one of the most common solutions for clinical bone defects^8^. However, there are some problems in the operation of the Masquelet technique. First, the size of the appropriate bone cement packing is difficult to control. If the induced membrane formation is too small, the bone connection formed by the graft bone will be too small, which will affect bone repair. According to Yin *et al*.^9^, the good effect of Masquelet technology is affected by the integrity of the induced membrane and the size of the induced membrane. Second, it is difficult to remove the filled bone cement during the second‐stage operation, and it is easy to damage the induction membrane when removing the bone cement, which may lead to reinfection after the operation. Zhou *et al*.^10^ analyzed the treatment of 44 cases of infectious bone defects. They found that the placement of bone cement fillings affected the size and integrity of the induction membrane. Using the Masquelet technique, it is difficult to control the size of the induction membrane during the operation. It is difficult to remove bone cement and damage‐inducing membrane increases the risk of infection.

Three‐dimensional printing technology, also known as rapid prototyping technology, provides high‐quality radiographic data based on the comparison of the results of a CT scan on the healthy side and the affected side, and uses computer technology to assist in design and manufacturing. Quickly and accurately directly generate the target model of any structure needed^11^. In recent years, three‐dimensional (3D) printing technology has become increasingly widely used in clinical practice, and has achieved satisfactory results, leading to new ideas for reconstruction of calcaneal defects^12^. Therefore, the present study reviewed the application of 3D printing technology‐assisted induction membrane technology to treat four patients with calcaneal defects. The purpose of this study is to: (i) introduce the surgical method of using 3D printing technology combined with induction membrane technology to treat calcaneal bone defects; (ii) improve the Masquelet technology and improve the quality of the formation of the induced membrane; and (iii) evaluate the clinical efficacy of the surgical plan.

## Methods

### Inclusion Criteria and Exclusion Criteria

This is a retrospective study of a collection of patients with calcaneal bone defects treated with 3D printing technology combined with induction membrane technology from January 2018 to April 2019.

The inclusion criteria were: (i) defect of the calcaneus caused by trauma; (ii) repair of soft tissue defect by skin flap transfer, 3D printing technology combined with induced membrane technology to treat patients with calcaneal bone defects; (iii) complete clinical and case data; and (iv) retrospective study.

The exclusion criteria were: (i) age <15 years or >60 years; (ii) history of foot surgery; and (iii) congenital ankle deformity and ankle deformity caused by previous injury.

### General Information

According to the inclusion and exclusion criteria, 3D printing combined with induced membrane technology was used to treat four patients with calcaneal bone defects from January 2018 to April 2019, including two men and two women. The patients were 22–52 years old, with an average age of 36 years. There were two cases of traffic accident injuries, one case of fall from height, and one case of crush injury. The bone defects of the four cases were 4 cm × 3 cm × 3 cm, 4 cm × 2 cm × 2 cm, 3 cm × 3 cm × 2 cm, and 3 cm × 2 cm × 1 cm; the combined skin defects were 12 cm × 7 cm, 10 cm × 6 cm, 9 cm × 5 cm, and 7cm × 6cm. Among them, there were three cases of infectious bone defects (two cases of *Escherichia coli* and one case of *Pseudomonas aeruginosa*).

### Three‐Dimensional Printing Personalized Bone Cement Shaping Device

**Fig. 1:**
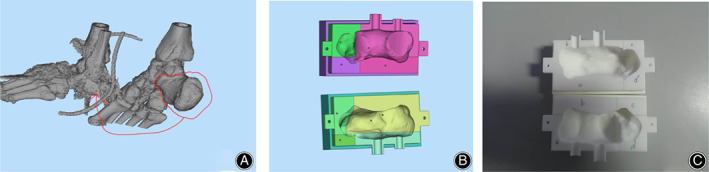
(A) Three‐dimensional printed model of the affected side and healthy side. (B) Mold design of the shaper. (C) Three‐dimensional printed mold of the shaper.

A 64‐slice spiral CT (Siemens, Germany) was used to scan the bone defect area and the corresponding healthy side, with a thickness of 1.0 mm. The data were saved in DICOM format, and we used the Mimics software to reconstruct the data in 3D (Fig.1A). The healthy side was used as a template to restore the virtual image of the defect and fit the data of the defect area to the data of the healthy side. We determined the anatomical landmark points and designed the digital model of the bone cement implant with the normal bone tissue from debridement, with 5 mm as the osteotomy plane; the model was saved in STL format. We imported the STL files into Materialise' 3‐matic software, starting from the osteotomy surface, and designed the model to wrap the mold. We used the Boolean calculation tool to obtain the cavity data of the bone cement modeler. Based on the principle that the mold can be easily released, the mold was divided into multiple modules as needed (Fig.1B). The module data was imported into a 3D printer with a filling rate of 50% and a layer thickness of 0.1 mm. It was printed with polylactic acid material and sterilized for use (Fig.1C).

### Surgical Methods

#### First‐Stage Surgery

**Fig. 2:**
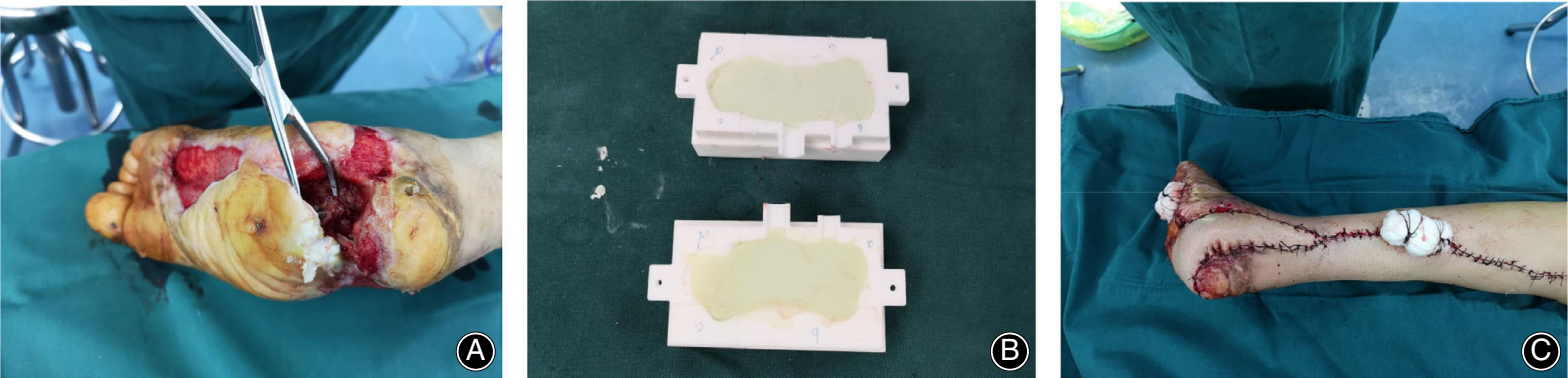
(A) We debrided the wound to the osteotomy surface and observed the seepage of fresh blood. (B) The shaper mold solidified the bone cement. (C) The lateral skin flap of the leg repaired the skin defect of the foot.

1. Place the patient in the lateral or decubitus position. Use either a combined spinal–epidural anesthesia or general anesthesia and operate under the control of a tourniquet.

2. Cut the skin and subcutaneous tissue layer by layer to fully expose the defect area; expand the wound to expose normal soft tissue, and remove dead bone and hardened bone until fresh blood leaks from the osteotomy surface (Fig.2A).

3. For infectious patients, take samples for susceptibility testing. The wounds need to be washed repeatedly with hydrogen peroxide and normal saline, and then washed with normal saline after soaking in iodophor.

4. Fully mix antibiotics (usually vancomycin) and bone cement powder and fill the molder module with antibiotic bone cement in a semi‐solidified state. After the antibiotic bone cement is completely solidified, remove the model. (Fig. 2B).

5. Implant the shaped antibiotic bone cement into the bone defect and use the internal (external) fixation device to fix the two after the two closely fit.

6. Repair the skin flap with local flap transposition or with a free thigh anterolateral flap (Fig. 2C).

7. Thoroughly flush the operation area, use negative pressure drainage, and suture the incision in layers.

#### Second‐Stage Surgery

**Fig. 3:**
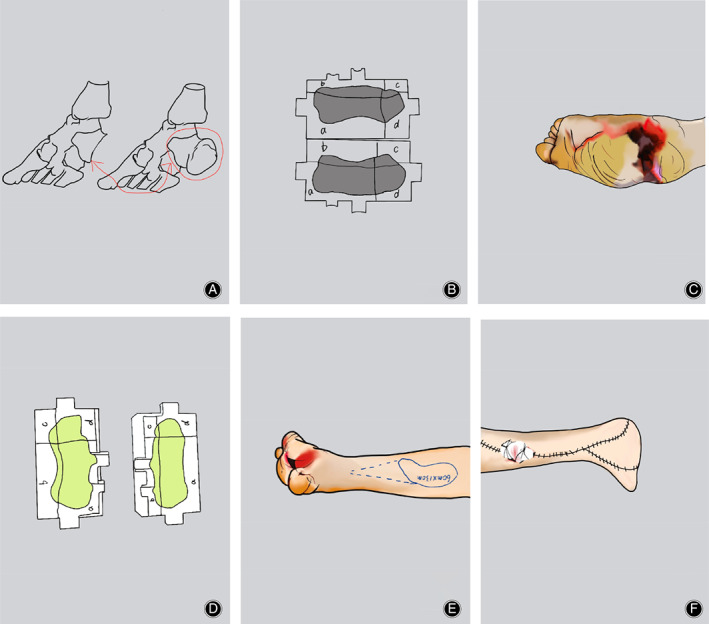
Key steps of surgery: (A) Mirror restoration of the defect of the calcaneus; (B) build the shaper model; (C) debride the bone defect area; (D) use a shaper to mold and solidify the bone cement into blocks and place the solidified bone cement into the bone defect area; (E) design a flap to cover the skin defect of the foot; and (F) transfer skin flaps to repair skin defects.

1. Place the patient in the lateral or decubitus position. Use either a spinal–epidural anesthesia or general anesthesia and operate under the control of a tourniquet.

2. Follow the original surgical incision and cut the skin layer by layer to expose the bone defect.

3. Cut the surface of the bone cement and remove the antibiotic bone cement completely. Clear the surrounding hardened area to promote blood circulation.

4. Take an appropriate amount of iliac bone trimmed into granules and implant it in the bone defect‐induced membrane and suture the induced membrane.

5. After thoroughly flushing the surgical area with normal saline, suture the wound layer by layer (Fig. 3).

### Postoperative Treatment

#### First‐Stage Surgery

Postoperative immobilization, raising the affected limb. Papaverine to prevent vasospasm. The blood supply of the skin flap should be observed every 2 h for 72 h after the operation. Removing the drainage tube after 48 h. The patient should be instructed to take the initiative to exercise the toes dorsiflexion function. Antibiotics are used according to the results of drug sensitivity, and infectious bone defects can extend the medication time. Two to three weeks after the operation, the patient can commence partial weight‐bearing activities.

#### First‐Stage Surgery

Observe the blood supply of the flap every 2 h for 24 h after the operation and remove the drainage tube after 48 h. Use routine treatments to prevent infection, swelling, and pain relief, and other treatments to prevent deep vein thrombosis. Instruct patients to present for review at 1, 2, 3, 6, and 12 months after surgery. Weight‐bearing is forbidden for 3 months after the operation. Gradually, weight‐bearing activities are carried out following the formation of callus after 3 months. During weight‐bearing activities, soft insoles should be used to cushion the pressure of the flap.

### Efficacy Evaluation Indicators

Curative effect indicators for the first stage include: (i) the matching degree of the intraoperative plastic bone cement and the bone end; and (ii) whether the bone cement needs to be adjusted. Curative effect indicators for the second stage include: (i) the quality of the induced membrane; (ii) whether the bone cement can be removed easily; (iii) bone defect healing time; and (iv) the Maryland Foot Score, which is used to evaluate foot function (the score includes pain [45 points] and function [gait, appearance, and mobility, 55 points]; the full score is 100 points, with a score of 90–100 points considered excellent, 75 to 89 points good, 50 to 74 points acceptable, and less than 50 points poor); and (v) the American Orthopedic Foot and Ankle Society (AOFAS) ankle–hind foot score was used to evaluate the ankle joint, including pain, function, gait, stability, and other aspects of assessment, with a score of 90–100 points considered excellent, 75 to 89 points good, 50 to 74 points acceptable, and less than 50 points poor.

## Results

### The Result of Intraoperative Bacterial Culture

During the first‐stage operation, multiple tissues were taken for bacterial culture. The results showed that there were two cases of *Escherichia coli* and one case of *Pseudomonas aeruginosa*. After debridement, four cases of calcaneal defect did not involve the calcaneal–talar joint and the calcaneocuboid joint.

### General Results

The operations of the four patients in this study were performed by the same surgical team.

#### First‐Stage Surgery

The operation time ranged from 120 to 200 min, with an average duration of 155 min; blood loss ranged from 500 to 1100 mL, with an average of 750 mL. During the operation, the tissues of the defect were taken for bacterial culture; the results show two cases of *Escherichia coli* and one case of *Pseudomonas aeruginosa*. When the bone cement is implanted into the bone defect area, the bone cement that is externally shaped by the shaper has a high matching degree with the bone end, and can completely fill the bone defect area without needing to adjust the bone cement again. Wound exudation occurred in one case after the operation, and the condition improved after enhanced dressing change. The rest of the wounds healed well, the skin flaps were alive, and there was no infection in the deep bone defect area.

#### Second‐Stage Surgery

The second‐stage surgery occurred 8–14 weeks after the first‐stage operation, with an average of 11.5 weeks; the operation time was 90 –160 min, with an average of 120 min; the bleeding volume was 300–700 mL, with an average of 475 mL. During the operation, it was confirm that the induction membrane was complete and the size was appropriate; the bone cement could be removed easily, with no obvious damage to the induced membrane. The wound healed well after the operation, and deep vein thrombosis did not occur.

All four patients in this group were followed up for 14 months (10–18 months). During the follow up, none of the foot flaps experienced necrosis or damage.

### Fracture Healing

The bone grafts of the four patients in this group all healed and the fractures healed well: X‐ray films showed continuous callus formation at the bone defect and no dead bones, and the continuity and integrity of the bones were acceptable. The clinical healing time was 4 months (range, 3–6 months).

### Maryland Foot Score

This group of patients walked with crutches at 14 weeks (range, 12–16 weeks) after the operation, and walked with full weight at 13 months (range, 9–15 months) after the operation. The Maryland foot score was 94 points (range, 88–98 points); three cases were excellent and one case was good.

### American Orthopedic Foot and Ankle Society Ankle–Hindfoot Score

**Fig. 4:**
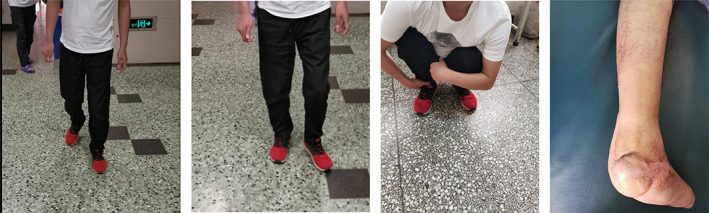
Appearance and function at the last follow‐up.

At 12 months postoperatively, the AOFAS ankle–hindfoot score was 92.8 points (range, 86–98 points); three cases were excellent and one case was good.(Fig. 4).

### Complications

There were no complications such as recurrence of infection, broken skin flap, loose fixation, bone resorption, or delayed healing in the four patients after the operation.

## Discussion

### Skin Flap Transfer to Repair Skin Defects and Three‐Dimensional Printing Combined with Masquelet Technology to Treat Calcaneal Bone Defects

For the four patients in this group, 3D printing mirror technology was used to print the model of the affected side and the healthy side before the operation. The osteotomy surface was determined by imaging data and CT scan data to provide data parameters for 3D printing of the defect on the affected side. The shape and size of the bone cement are based on the shape and size of the defect of the calcaneus on the affected side. Accordingly, 3D printing was used to produce a plastic mold that would suit the shape and size of the bone cement. The quality and size of the induced membrane can be managed by controlling the implantation of bone cement. In the first stage of surgery, skin flap transfer was used to repair foot skin defects, providing appropriate conditions for the application of Masquelet technology. In a meta‐analysis, Morelli *et al*.^13^ also explained the importance of soft tissue reconstruction for Masquelet technology; the bone defect area was debrided according to the osteotomy before the operation, and fresh blood was observed leaking out. The bone cement molded by the mold was placed into the bone defect area in pieces. The bone cement had a high degree of matching with the bone defect part, and there was no need to adjust the shape and size.

In the second stage of surgery, it was confirmed that the formed induction membrane was of appropriate size, and the bone cement could be removed in pieces without significant damage to the induction membrane. Patients avoided weight‐bearing for 3 months after bone grafting and could gradually bear weight when the calcaneal bone had healed. The bone grafts healed in all four patients, and bone trabeculae gradually appeared after normal walking, realizing the reconstruction of calcaneal bone. According to the AOFAS score results, three cases were excellent and one case was good, which also confirmed the successful recovery of the calcaneus physiological function. After the follow up, there was no necrosis or rupture of the skin flap. The main reason for this was the immobilization for 3 months after surgery, which not only prevented the small bones from being easily deformed but also affected the maintenance of the shaped arch as well as the function. It also prevented small bones from applying different degrees of pressure on the skin flap, reducing the impact on the survival and integrity of the skin flap. In addition, the soft insole cushioned the pressure that the skin flap withstood after gradually bearing weight.

**Fig. 5:**
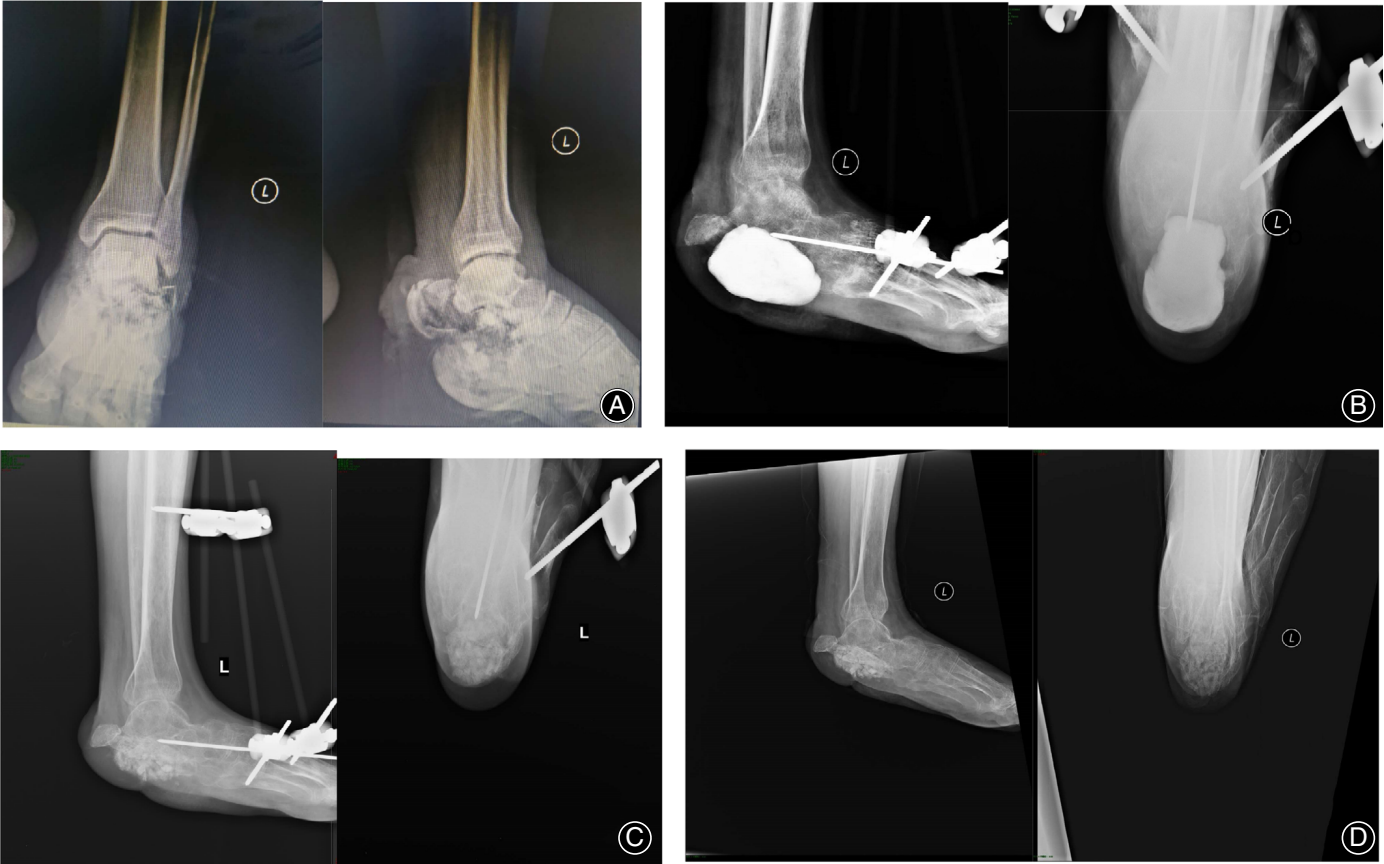
(A) X‐ray film before operation. (B) X‐ray film after bone cement implantation. (C) X‐ray film after bone grafting. (D) X‐ray film for the last follow up.

Soft tissue defects were repaired in all four patients through skin flap transfer and 3D printing technology combined with Masquelet technology to treat calcaneal bone defects. Good clinical results were achieved in all patients (Fig. 5).

### Advantages of Three‐Dimensional Printing Technology Combined with Induced Membrane Technology in the Treatment of Calcaneal Defects

The traditional Masquelet technology can not accurately grasp the size and shape of bone defect in bone cement filling. The production of antibiotic bone cement can be divided into *in vivo* molding and *in vitro* molding. Traditional *in vivo* molding fills the bone defect with bone cement before it solidifies and includes the fractured end so that it naturally solidifies. During the setting of bone cement, heat is released, and adjacent bones and adjacent tissues are also damaged. During the second stage of the operation, it was found that the bone cement was firmly connected to the bone end, and can easily damage the induced membrane and the bone tissue of the bone cement–bone interface when it is taken out. It affects fracture healing after surgery and can also cause other complications. To overcome the shortcomings of bone cement *in vivo* molding, some scholars have used bone cement to shape *in vitro* ^14^. However, there are many types of bone defects in clinical practice, and it is difficult to comprehensively and accurately reflect bone defect information using imaging data. The size and shape of the bone cement implant can be subjectively controlled by the doctor. Therefore, the implantation of antibiotic bone cement is one of the key components of this technology.

According to Simpson *et al*.^15^, when the debridement exceeds the normal bone tissue by 5 mm, it can effectively reduce the incidence of later infection. This provides a theoretical basis for determining the osteotomy surface that needs debridement before surgery, thereby determining the shape and size of the bone defect area, and also provides theoretical support for the application of 3D printing technology. In this study, 3D printing technology was used to reconstruct the calcaneal defect area in three dimensions, and the bone cement shaping device was printed according to the calcaneal defect area. Using the method of external shaping, the thermal damage to bone and soft tissues caused by the internal shaping of the bone cement is avoided, and the bone cement and the bone end are not connected too firmly. Using the multi‐module design also avoids the difficulty of removing large pieces of bone cement and damaging the induced membrane, thus ensuring the quality of the induced membrane. In addition, the shape and size of the bone cement and the calcaneus are maintained at the same height, which not only conforms to the biomechanical conduction of the defect area but also ensures the formation of an appropriately sized induction membrane. Finally, the production of bone cement is no longer subjectively influenced by the doctor, and it will also reduce the formation of bubbles and delamination in the bone cement filling process.

Therefore, 3D printing technology combined with Masquelet technology to treat calcaneal bone defects can ensure the quality and size of the induced membrane and has the advantages of simple and efficient technology and biomechanics. There were no complications after bone grafting, and at the last follow up, the patient did not complain of pain symptoms and the functional recovery was good; these results were related to the formation of high‐quality induction membranes.

### Shortcomings of this Study

First, due to the low incidence of calcaneal bone defects combined with soft tissue defects, the sample size of this study is small. Using a larger sample size is necessary to continue to observe the clinical efficacy and analyze other problems that may occur during the operation. Second, the lack of comparative research. It is necessary to compare with those for traditional vascular graft or bone transfer technology to objectively analyze the clinical efficacy of 3D printing combined with Masquelet technology in treating calcaneal bone defects. Third, when the first operation is unsuccessful due to the contracture of the skin flap, to ensure that the molded bone cement can fill the defect area, the model of the second operation should be appropriately reduced. Finally, the patient may have damage to the ligaments and other tissues of the foot, which affects the treatment effect and needs to be resolved.

### Conclusion

In short, this study provides new ideas and methods for the treatment of calcaneal bone defects. Skin flap transfer to repair soft tissue defects and 3D printing combined with Masquelet technology to treat calcaneal bone defects has satisfactory results.
